# Use of Monoclonal Antibody to Treat COVID-19 in Children and Adolescents: Risk of Abuse of Prescription and Exacerbation of Health Inequalities

**DOI:** 10.3390/ph14070673

**Published:** 2021-07-15

**Authors:** Susanna Esposito, Stefano Zona, Andrea Pession, Lorenzo Iughetti, Giovanni Battista Migliori, Nicola Principi

**Affiliations:** 1 Paediatric Clinic, University Hospital, Department of Medicine and Surgery, University of Parma, 43126 Parma, Italy; 2 AUSL Modena, 41121 Modena, Italy; s.zona@ausl.mo.it; 3 Paediatric Clinic, IRCCS Azienda Ospedaliero-Universitaria di Bologna, University of Bologna, 40138 Bologna, Italy; andrea.pession@unibo.it; 4 Paediatric Unit, Department of Medical and Surgical Sciences of Mothers, Children and Adults, University of Modena and Reggio Emilia, 41125 Modena, Italy; lorenzo.iughetti@unimore.it; 5 Servizio di Epidemiologia Clinica delle Malattie Respiratorie, Istituti Clinici Scientifici Maugeri IRCCS, 21049 Tradate, Italy; gbmigliori@gmail.com; 6 Università degli Studi di Milano, 20122 Milan, Italy; nicola.principi@unimi.it

**Keywords:** COVID-19, passive immunotherapy, pediatric infectious diseases, SARS-CoV-2, monoclonal antibody

## Abstract

Monoclonal antibodies (mAbs) that neutralize SARS-CoV-2 in infected patients are a new class of antiviral agents approved as a type of passive immunotherapy. They should be administered to adults and children (≥12 years old, weighing ≥ 40 kg) with SARS-CoV-2 positivity, and who are suffering from a chronic underlying disease and are at risk of severe COVID-19 and/or hospitalization. The aim of this manuscript is to discuss the benefit-to-risk of mAb therapy to treat COVID-19 in pediatric age, according to current reports. A problem is that the authorization for mAbs use in children was given without studies previously evaluating the efficacy, safety and tolerability of mAbs in pediatric patients. Moreover, although the total number of children with chronic severe underlying disease is not marginal, the risk of severe COVID-19 in pediatric age is significantly reduced than in adults and the role of chronic underlying disease as a risk factor of severe COVID-19 development in pediatric patients is far from being precisely defined. In addition, criteria presently suggested for use of mAbs in children and adolescents are very broad and may cause individual clinicians or institutions to recommend these agents on a case-by-case basis, with an abuse in mAbs prescriptions and an exacerbation of health inequalities while resources are scarce. Several questions need to be addressed before their routine use in clinical practice, including what is their associated benefit-to-risk ratio in children and adolescents, who are the patients that could really have benefit from their use, and if there is any interference of mAb therapy on recommended vaccines. While we wait for answers to these questions from well-conducted research, an effective and safe COVID-19 vaccine for vulnerable pediatric patients remains the best strategy to prevent COVID-19 and represents the priority for public health policies.

## 1. Background

In the current coronavirus disease 2019 (COVID-19) pandemic, several prophylactic and therapeutic approaches are being developed or repurposed to combat COVID-19. Monoclonal antibodies (mAbs) that can neutralize the severe acute respiratory syndrome coronavirus 2 (SARS-CoV-2) in infected patients are a novel class of antiviral intervention for which the US Food and Drug Administration (FDA) and European Medicines Agency (EMA) provided Emergency Use Authorization (EUA) [1,2]. All mAbs are approved as a type of passive immunotherapy to minimize virulence for the treatment of non-hospitalized patients with mild-to-moderate COVID-19. They should be administered to adults and children (≥12 years old, weighing ≥ 40 kg) with positive results of direct SARS-CoV-2 testing, and who are suffering from a chronic underlying disease that may favor progression to severe COVID-19 and/or hospitalization [1,2]. Risk factors for severe COVID-19 may include but are not limited to obesity; cardiovascular disease, including hypertension; chronic lung disease, including asthma; type 1 or type 2 diabetes mellitus; chronic kidney disease, including those on dialysis, chronic liver disease; immunosuppressed, based on prescriber’s assessment (i.e., cancer treatment, bone marrow or organ transplantation, immune deficiencies, HIV sickle cell anaemia, thalassaemia, and prolonged use of immune-weakening medications) [3]. However, looking at EUAs, the logic of considering older children as adults could be debated. The aim of this manuscript is to discuss the benefit-to-risk of mAb therapy to treat COVID-19 in pediatric age according to current reports.

## 2. Monoclonal Antibodies against SARS-CoV-2: Pharmacological Considerations

SARS-CoV-2-neutralizing mAbs primarily bind the trimeric spike (S) glycoproteins on the viral surface that permit entry into host cells. The S protein has two functional subunits that permit cell attachment (the S1 subunit, with four core domains S1A through S1D) and fusion of the viral and cellular membrane (the S2 subunit). Potent neutralizing mAbs often neutralize the receptor in S1, disabling receptor interactions [4]. The spike proteins of SARS-CoV-2 (SARS2-S) and SARS-CoV (SARS-S) are 77.5% identical by primary amino acid sequence, are structurally very similar and commonly bind the human angiotensin-converting enzyme 2 (ACE2) protein through their S1B domain [5,6]. Receptor interaction is known to cause irreversible conformational modifications in coronavirus spike proteins enabling membrane fusion [7]. MAbs targeting the S protein can prevent SARS-CoV-2 infection, alleviate symptoms and limit progression to severe disease in patients with mild to moderate COVID-19, especially in those without an endogenous antibody response.

The first mAbs authorized against SARS-CoV-2 were bamlanivimab as a monotherapy, and bamlanivimab associated with etesevimab or casirivimab with imdevimab as a combination therapy, but new products are in an advanced stage of evaluation [8]. However, the EUA of bamlanivimab was revoked by the FDA after few months because in vitro studies had shown that multiple independent viral escape mutants could be generated to each of the individual mAbs tested, whereas viral escape mutants were not selected in the presence of non-competing mAb combinations [9]. On the other hand, a clinical evaluation had shown that after the introduction of bamlanivimab, approximately 20% of isolates sequenced in the USA from infected persons were reported as lineages resistant to this mAb. On the contrary, combination therapy was effective without any significant adverse events. When bamlanivimab was associated with etesevimab, a significant decrease in patient’s viral load as well as in COVID-19-related hospitalizations and deaths was observed without any increase in resistant variants and with a number of patients needed to treat to observe benefits of 21 [8]. Similarly, compared to placebo, therapy with casirivimab and imdevimab was significantly more effective, reducing viral load in adults, and also diminishing COVID-19-related medically attended visits and hospitalizations, with a number of patients needed to treat of 17 and 50, respectively [10].

More than 50 mAb-related clinical trials are being conducted in different countries around the world. However, as most are in a very early stage, there has not been enough data collected to adequately evaluate them for EUA by the FDA and EMA [1,2]. Although mAbs are one of the fastest-growing drug classes in recent years, their precise mechanism of action is yet unknown. Any outcome with therapeutic mAb is related to several factors. Important factors include antigen cell-surface density, tissue distribution, specificity, avidity, and isotype [11]. A homogeneous group of patients with the same line(s) of therapy or severity or stage of disease progression, and those receiving first-line therapy are the ideal candidate for mAb therapies. The reason for the slow speed in developing mAbs includes unreasonable costing for research and development, especially when compared with small molecule drugs and vaccines [12]. Additionally, the complexity and ambiguity of viruses as associated with their rapid mutation make it difficult for researchers to develop effective and long-lasting mAb therapy [13]. For all these reasons, “target specificity” (i.e., optimum timing of therapy for a specific population with a specific disease) is needed for the approval of mAbs use.

## 3. Do the Benefits of Monoclonal Antibody (mAbs) Treatment for COVID-19 in Children and Adolescents Really Outweigh the Risks?

Theoretically, a great number of patients could benefit from mAb administration; particularly adults, that due to disease or age, are prone to develop severe COVID-19 [1,2]. Presently, virus circulation remains high and, due to the unknown duration of protection in vaccinated subjects, the total number of patients with risk factors still susceptible to SARS-CoV-2 infection remains relevant. However, to be effective, mAbs should be administered as soon as possible and this means that for a great number of subjects an accurate and continual monitoring of clinical conditions to identify the first clinical manifestations of SARS-CoV-2 infection should be planned [1,2]. In suspected cases, a nasopharyngeal swab must be obtained and processed, with positive cases transferred to the hospital for mAb administration. A complex organization including at least telemedical visits and laboratory tests must be planned with relevant economic costs. All of these problems can be overcome when the positive effects of mAb use are considered.

Despite these positive results, administration of mAbs to children and adolescents should be carefully evaluated and criteria for pediatric use of mAbs re-considered by health authorities in the official recommendations. A problem is that the authorization for mAb use in children was given without studies previously evaluating the efficacy, safety and tolerability of mAbs in pediatric patients. Moreover, although the total number of children with chronic severe underlying disease is not marginal, the risk of severe COVID-19 in pediatric age is significantly lower than in adults [14,15] and the role of a chronic underlying disease as a risk factor of severe COVID-19 development in pediatric patients is far from being precisely defined [16]. When infected, pediatric patients generally are asymptomatic or with mild disease. A recent report showed that in the USA, on 22 April 2021, only 0.1–1.9% of all child COVID-19 cases were hospitalized, pediatric COVID-19 corresponded to 1.2–2.9% of hospitalizations associated with the disease and child mortality was 0.00–0.1921% of all COVID-19 deaths [17]. Few pediatric cases were admitted to the pediatric intensive care unit (PICU) as well as required respiratory support with mechanical ventilation and additional life-saving interventions. A multinational, multicenter cohort study performed in Europe demonstrated that among 582 SARS-CoV-2 positive children and a median age of 5.0 years, 62% were hospitalized, 8% were admitted to the PICU, 5% showed radiological findings suggestive of acute respiratory distress syndrome and 4% were intubated and mechanically ventilated [18]. Interestingly, there are no data on chronic diseases associated with an increased risk of severe disease. However, the importance of underlying diseases was found to be quite different in several studies, suggesting that differences in criteria for enrollment and evaluation could have been focused on final results rather than the underlying disease and its severity. Similar data were described in a European research study in which 48 children with COVID-19 requiring ventilation were analyzed [19]. In Italy, when a total of 168 pediatric patients initially evaluated in the outpatient setting were analyzed, no difference in hospitalization rate between patients with and without co-morbidities was observed [20]. Similarly, studies carried out in children receiving immunosuppressive treatment for various indications have shown that these patients do not suffer from severe COVID-19 more frequently than healthy children [21,22]. On the other hand, it cannot be excluded that in pediatrics only some of the underlying diseases considered risk factors for severe COVID-19 in adults can play a role. In New York, obese children but not immunocompromised patients had a severe COVID-19 [23]. In the UK, pediatric patients presenting with pre-existing COVID-19 vulnerable chronic diseases were not found to have an increased risk of either contracting COVID-19 or severe complications, with the exclusion of those undergoing chemotherapy [24].

In addition, identification of pediatric cases for whom mAbs are authorized can be difficult and require very complicated and expensive monitoring. A study planned to show the frequency of symptoms compatible with SARS-CoV-2 infection in pediatric patients with immunodeficiency has shown that despite 67.4% of study patients reporting one or more symptoms suggestive of suspected SARS-CoV-2 infection, of the 110 who were tested for virus none was positive for SARS-CoV-2 [25]. These results suggested an absence of symptom specificity in these patients, showing that symptoms of SARS-CoV-2 infection overlapped with those of chronic disease exacerbations. Consequently, in order to diagnose early COVID-19 to administer mAbs, systematic monitoring of all the patients with chronic underlying disease should be planned, with enormous efforts, costs, and poor final results. Moreover, as evidenced in this study, continuous monitoring could lead patients/parents to remain very anxious, demonstrating the pressing need to clearly define and communicate SARS-CoV-2 risk in children and young people. In the absence of data suggesting that specific conditions increase the risk of disease severity, it is mandatory to be very careful to conclude that there are significant benefits with the routine use of mAbs in children with any type of chronic underlying diseases [26]. These treatments are expensive, difficult to make because the administration is intravenous, and there is a risk of the development of drug-resistant variants.

Finally, there is limited published evidence on the coadministration of mAbs with pediatric vaccines. The mechanism of action of mAbs might provide insights into the potential for an anti-viral mAb to interfere with the immune response to a vaccine [27]. MAbs for treatment of COVID-19 are antibodies which exhibit high potency and expected specificity against viral pathogens and are engineered to further enhance their in vivo functions. MAb–vaccine interaction studies are generally not required by regulatory authorities to support licensure indicating the lack of necessity or clinical relevance of such evaluations. This is reflected in FDA or EMA regulatory guidelines. To date, the conduct of mAb–vaccine interference studies is limited to mAbs with an internal target involved in immune function to assess if the mAb would alter the function of the immune system and affect the response to vaccinations in the patient population [27]. Moreover, there are limited country-specific guidelines focusing on the coadministration of antibodies and vaccines. According to the USA and UK guidelines, live viral vaccines should be administered at least three weeks before or three to 11 months after an injection of intravenous immunoglobulin depending on the dose [27]. While specific studies investigating the coadministration of anti-viral mAbs with vaccines have not been performed, concerns could be raised. This means that the use of mAbs for COVID-19 in pediatric patients with chronic diseases could lead to a delay in vaccination, with a reduction in coverage for routine vaccines in the most fragile pediatric population. These concerns can have important implications in a historic moment such as this in which vaccination coverage has dropped significantly because parents skipped the vaccine appointment of their sons as they were afraid of SARS-CoV-2, or vaccination centers postponed the appointments because they were closed [28,29]. On the other hand, COVID-19 vaccines could actually represent the best option for vulnerable children and adolescents because of their cost-effectiveness [30,31], and the use of mAbs for COVID-19 treatment could negatively affect the immune response to the COVID-19 vaccines.

## 4. Conclusions

The criteria currently used for mAb therapy against SARS-CoV-2 are very broad and allow individual clinicians or institutions to use these agents on a case-by-case basis, adding to abuse in mAbs prescription and an exacerbation of health inequalities while resources are scarce. Several questions need to be addressed before their routine use in pediatric clinical practice, including what their associated benefit-to-risk ratio in children and adolescents is, who are the patients that could really have benefit from their use, and if there is an interference with mAb therapy on recommended vaccines. While we wait for answers to these questions from well-conducted research, an effective and safe COVID-19 vaccine for vulnerable pediatric patients remains the best strategy to prevent COVID-19 and represents the priority for public health.

## Data Availability

Data sharing not applicable. The text was written based on findings reported in the mentioned references.

## References

[1] U.S. Food & Drug Administration Coronavirus (COVID-19) Update: FDA Authorizes Monoclonal Antibody for Treatment of COVID-19. https://www.fda.gov/news-events/press-announcements/coronavirus-covid-19-update-fda-authorizes-monoclonal-antibody-treatment-covid-19.

[2] European Medicines Agency EMA Reviewing Data on Monoclonal Antibody Use for COVID-19. https://www.ema.europa.eu/en/news/ema-reviewing-data-monoclonal-antibody-use-covid-19.

[3] Gao Y.D., Ding M., Dong X., Zhang J.J., Kursat Azkur A., Azkur D., Gan H., Sun Y.L., Fu W., Li W. (2021). Risk factors for severe and critically ill COVID-19 patients: A review. Allergy.

[4] Dejnirattisai W., Zhou D., Supasa P., Liu C., Mentzer A.J., Ginn H.M., Zhao Y., Duyvesteyn H.M.E., Tuekprakhon A., Nutalai R. (2021). Antibody evasion by the P.1 strain of SARS-CoV-2. Cell.

[5] Collier D.A., De Marco A., Ferreira I.A.T.M., Meng B., Datir R.P., Walls A.C., Kemp S.A., Bassi J., Pinto D., Silacci-Fregni C. (2021). Sensitivity of SARS-CoV-2 B.1.1.7 to mRNA vaccine-elicited antibodies. Nature.

[6] Mei M., Tan X. (2021). Current Strategies of Antiviral Drug Discovery for COVID-19. Front. Mol. Biosci..

[7] Passariello M., Gentile C., Ferrucci V., Sasso E., Vetrei C., Fusco G., Viscardi M., Brandi S., Cerino P., Zambrano N. (2021). Novel human neutralizing mAbs specific for Spike-RBD of SARS-CoV-2. Sci. Rep..

[8] Taylor P.C., Adams A.C., Hufford M.M. (2021). de la Torre, I.; Winthrop, K.; Gottlieb, R.L. Neutralizing monoclonal antibodies for treatment of COVID-19. Nat. Rev. Immunol..

[9] Baum A., Fulton B.O., Wloga E., Copin R., Pascal K.E., Russo V., Giordano S., Lanza K., Negron N., Ni M. (2020). Antibody cocktail to SARS-CoV-2 spike protein prevents rapid mutational escape seen with individual antibodies. Science.

[10] Regeneron Pharmaceuticals Inc Regeneron’s COVID-19 Outpatient Trial Prospectively Demonstrates that REGN-COV2 Antibody Cocktail Significantly Reduced Virus Levels and Need for Further Medical Attention. https://investor.regeneron.com/news-releases/news-release-details/regenerons-covid-19-outpatient-trial-prospectively-demonstrates.

[11] Shanmugaraj B., Siriwattananon K., Wangkanont K., Phoolcharoen W. (2020). Perspectives on monoclonal antibody therapy as potential therapeutic intervention for Coronavirus disease-19 (COVID-19). Asian Pac. J. Allergy Immunol..

[12] Deb P., Molla M.M.A., Rahman K.S.U. (2021). An update to monoclonal antibody as therapeutic option against COVID-19. Biosaf Health.

[13] Cohen M.S., Wohl D.A., Fischer W.A., Smith D.J., Eron J.J. (2021). Outpatient Treatment of SARS-CoV-2 Infection to Prevent COVID-19 Progression. Clin. Infect. Dis..

[14] Cusenza F., Davino G., D’alvano T., Argentiero A., Fainardi V., Pisi G., Principi N., Esposito S. (2021). Silence of the Lambs: The Immunological and Molecular Mechanisms of COVID-19 in Children in Comparison with Adults. Microorganisms.

[15] Esposito S., Marchetti F., Lanari M., Caramelli F., De Fanti A., Vergine G., Iughetti L., Fornaro M., Suppiej A., Zona S. (2021). COVID-19 Management in the Pediatric Age: Consensus Document of the COVID-19 Working Group in Paediatrics of the Emilia-Romagna Region (RE-CO-Ped), Italy. Int. J. Environ. Res. Public Health.

[16] Esposito S., Caramelli F., Principi N. (2021). What are the risk factors for admission to the pediatric intensive unit among pediatric patients with COVID-19?. Ital. J. Pediatr..

[17] American Academy of Pediatrics Children and COVID-19: State-Level Data Report. https://downloads.aap.org/AAP/PDF/AAP%20and%20CHA%20-%20Children%20and%20COVID-19%20State%20Data%20Report%201.28.21%20FINAL.pdf.

[18] Williams N., Radia T., Harman K., Agrawal P., Cook J., Gupta A. (2021). COVID-19 Severe acute respiratory syndrome coronavirus 2 (SARS-CoV-2) infection in children and adolescents: A systematic review of critically unwell children and the association with underlying comorbidities. Eur. J. Pediatr..

[19] Götzinger F., Santiago-García B., Noguera-Julián A., Lanaspa M., Lancella L., Carducci F.I.C., Gabrovska N., Velizarova S., Prunk P., Osterman V. (2020). COVID-19 in children and adolescents in Europe: A multinational, multicentre cohort study. Lancet Child Adolesc. Health.

[20] De Jacobis I.T., Vona R., Cittadini C., Marchesi A., Cursi L., Gambardella L., Villani A., Straface E. (2021). Clinical characteristics of children infected with SARS-CoV-2 in Italy. Ital. J. Pediatr..

[21] Marlais M., Wlodkowski T., Vivarelli M., Pape L., Tönshoff B., Schaefer F., Tullus K. (2020). The severity of COVID-19 in children on immunosuppressive medication. Lancet Child Adolesc. Health.

[22] Turner D., Huang Y., Martín-de-Carpi J., Aloi M., Focht G., Kang B., Zhou Y., Sanchez C., Kappelman M.D., Uhlig H.H. (2020). Coronavirus Disease 2019 and Paediatric Inflammatory Bowel Diseases: Global Experience and Provisional Guidance (March 2020) from the Paediatric IBD Porto Group of European Society of Paediatric Gastroenterology, Hepatology, and Nutrition. J. Pediatr. Gastroenterol. Nutr..

[23] Zachariah P., Johnson C.L., Halabi K.C., Ahn D., Sen A.I., Fischer A., Banker S.L., Giordano M., Manice C.S., Diamond R. (2020). Epidemiology, Clinical Features, and Disease Severity in Patients with Coronavirus Disease 2019 (COVID-19) in a Children’s Hospital in New York City, New York. JAMA Pediatr..

[24] Issitt R., Booth J., Bryant W., Spiridou A., Taylor A.M., du Pré P. (2020). Children with COVID-19 at a specialist centre: Initial experience and outcome. Lancet Child Adolesc. Health.

[25] Shaunak M., Patel R., Driessens C., Mills L., Leahy A., Gbesemete D., Owens D.R., Lucas J.S., Faust S.N., de Graaf H. (2021). COVID-19 symptom surveillance in immunocompromised children and young people in the UK: A prospective observational cohort study. BMJ Open.

[26] Wolf J., Abzug M.J., Wattier R.L., Sue P.K., Vora S.B., Zachariah P., Dulek D.E., Waghmare A., Olivero R., Downes K.J. (2021). Initial Guidance on Use of Monoclonal Antibody Therapy for Treatment of COVID-19 in Children and Adolescents. J. Pediatr. Infect. Dis. Soc..

[27] Walker L.M., Burton D.R. (2018). Passive immunotherapy of viral infections: ’super-antibodies’ enter the fray. Nat. Rev. Immunol..

[28] Russo R., Bozzola E., Palma P., Corsello G., Villani A. (2021). Pediatric routine vaccinations in the COVID 19 lockdown period: The survey of the Italian Pediatric Society. Ital. J. Pediatr..

[29] Bonanni P., Angelillo I.F., Villani A., Biasci P., Scotti S., Russo R., Maio T., Vitali Rosati G., Barretta M., Bozzola E. (2021). Maintain and increase vaccination coverage in children, adolescents, adults and elderly people: Let’s avoid adding epidemics to the pandemic: Appeal from the Board of the Vaccination Calendar for Life in Italy. Vaccine.

[30] Principi N., Esposito S. (2021). Why It Is Important to Develop an Effective and Safe Pediatric COVID-19 Vaccine. Vaccines.

[31] Mahase E. (2021). Covid vaccine could be rolled out to children by autumn. BMJ.

